# Thrombus Composition and the Evolving Role of Tenecteplase in Acute Ischemic Stroke

**DOI:** 10.3390/jcm14248675

**Published:** 2025-12-07

**Authors:** Senta Frol, Matija Zupan

**Affiliations:** 1 Department of Vascular Neurology, University Medical Centre Ljubljana, Zaloška cesta 2, 1000 Ljubljana, Slovenia; 2 Faculty of Medicine, University of Ljubljana, Vrazov trg 2, 1000 Ljubljana, Slovenia

**Keywords:** acute ischemic stroke, combination therapies, tenecteplase, thrombus composition, treatment

## Abstract

Acute ischemic stroke (AIS) is a leading cause of disability and death worldwide, requiring rapid reperfusion to minimize damage. Current treatments, including intravenous thrombolysis (IVT) with alteplase (rt-PA) and mechanical thrombectomy (MT), face limitations such as thrombolysis resistance, dosing complexity, and reduced efficacy in large vessel occlusions (LVOs) or fibrin-rich clots. Tenecteplase (TNK), a bioengineered thrombolytic agent with superior pharmacokinetics, simplified administration, and higher fibrin specificity, offers promising advantages over rt-PA, including potential synergy with MT and efficacy against resistant thrombi. Direct oral anticoagulants (DOACs) further complicate AIS management, but evidence suggests that DOAC-treated patients may experience better thrombolysis outcomes due to distinct thrombus characteristics. Advances in imaging now enable precise visualization of vessel occlusion and treatment effects, opening opportunities to refine therapies. Combination approaches targeting fibrin thrombus components may enhance thrombolysis and improve outcomes in resistant cases. Future research should explore TNK’s role in intra-arterial (IA) applications, combination therapies, and its interaction with MT to optimize reperfusion strategies. TNK’s simplified use and promising efficacy position it as a potential breakthrough in AIS management, with the potential to improve functional recovery and reduce treatment complexity.

## 1. Introduction

Acute ischemic stroke (AIS), the most prevalent subtype of stroke, remains a leading global cause of disability and mortality. It arises from thromboembolic occlusion of cerebral arteries, resulting in ischemic injury caused by reduced blood flow. Rapid and effective treatments, such as intravenous thrombolysis (IVT) and mechanical thrombectomy (MT), are essential to restore cerebral perfusion and minimize neuronal damage. The efficacy of these interventions is closely tied to thrombus characteristics and the timeliness of reperfusion, highlighting the importance of early diagnosis and prompt intervention to optimize patient outcomes [1,2].

The primary aim of our paper is to propose a framework for precision reperfusion treatment, with the prospect of tailoring reperfusion treatment to a putative composition of AIS thrombi—and hence their resistance to reperfusion treatment—based on their radiological appearance. Firstly, we discuss current approaches in AIS management, the relation of thrombus composition to the resistance to reperfusion treatments, and challenges in AIS patients currently on direct oral anticoagulants (DOACs). Further, we highlight the advantages of IVT with tenecteplase (TNK) over alteplase (rt-PA), and intra-arterial (IA) TNK use as an add-on therapy after MT. We round off our discussion by the potential of adjunctive therapies targeting specific thrombus components and active processes other than fibrin to enhance thrombolysis efficacy.

## 2. Current Approaches in Acute Ischemic Stroke Management

IVT with rt-PA has served as the cornerstone of AIS treatment for nearly three decades. However, rt-PA’s short half-life necessitates a complex dosing regimen comprising an initial bolus followed by continuous infusion. This introduces practical challenges, including potential delays, dosing interruptions and inaccuracies, and reliance on trained healthcare personnel [3,4]. Such complexities are further exacerbated during interfacility transfers, where treatment delays may negatively affect outcomes. Additionally, rt-PA carries a risk of symptomatic intracerebral hemorrhage (sICH), and its efficacy is limited, particularly in large vessel occlusions (LVOs).

For patients with LVOs, MT is the recommended treatment approach [2]. Bridging therapy, combining IVT with MT, has shown benefits by softening thrombi, facilitating mechanical retrieval, and reducing complications such as the no-reflow phenomenon [5]. However, rt-PA fails in over 50% of cases overall due to thrombolysis resistance [6], underscoring the urgent need for novel therapeutic strategies to overcome these limitations.

## 3. Thrombus Composition and Resistance to Treatment

Thrombus composition plays a pivotal role in determining the success of both IVT and MT. Stroke thrombi exhibit variability in their structural and biochemical properties, comprising fibrin, red blood cells (RBCs), platelets, neutrophil extracellular traps (NETs), extracellular deoxyribonucleic acid (DNA), and von Willebrand factor (VWF). Furthermore, molecules such as plasminogen activator inhibitor-1 (PAI-1) play a pivotal role in thrombus resistance to treatment. The site and mechanism of thrombus formation (e.g., cardioembolic vs. large artery atherosclerosis) influence its composition and structure [7]. Retrievable thrombi allow histological study, offering clues to stroke etiology [8]. In embolic stroke of undetermined source, thrombus characteristics may help reclassify likely mechanisms (e.g., occult cardioembolism) and direct secondary prevention strategies [9].

RBC-rich thrombi—often but not exclusively associated with cardioembolic strokes—are characterized by loosely organized fibrin networks, are more responsive to both IVT and MT due to their less compact structure, which permits better penetration of thrombolytic agents and easier retrieval in MT [10]. Imaging features such as hyperdense vessel sign on non-contrast computerized tomography (CT) and blooming artifact on magnetic resonance imaging (MRI) gradient-recalled echo (GRE)/susceptibility-weighted imaging (SWI) correlate with RBC-rich thrombi [11]. Measures like clot perviousness, i.e., clot permeability for contrast medium on CT angiography (CTA), and emerging radiomic features (texture, heterogeneity) may provide noninvasive predictions of thrombus properties [12]. Advanced tools (e.g., dual-energy CT, positron emission tomography (PET) radiotracers) are under investigation to distinguish further clot composition in vivo [13].

In contrast, thrombi that are fibrin- and platelet-dominant, with NETs and DNA interlaced, are often—but not exclusively—associated with atherothrombotic strokes, and are more resistant to treatment due to their dense, compact structure, resulting in increased recanalization maneuvers, longer procedure time, risk of vessel injury and distal embolization, and less favorable clinical outcomes [14]. These thrombi have lower density on CT and exhibit less prominent susceptibility on MRI. The tight structure of these thrombi limits the penetration of thrombolytic agents and hampers their effectiveness [15]. Furthermore, thrombi with associated calcification and cholesterol crystals, such as those found in atherothrombotic occlusions on pre-existent plaques, impede mechanical retrieval. Calcified thrombi exhibit a typical calcified appearance on CT with round or ovoid shape and higher attenuation compared to RBC-rich thrombi. Conversely, cholesterol-rich thrombi are hypodense on CT. Histopathological studies support that higher RBC fractions correlate with better recanalization success; conversely, more organized, dense, or high-leukocyte clots are associated with poorer outcomes [14]. Table 1 summarizes thrombus composition, imaging correlates and treatment response in AIS.

## 4. Challenges and Opportunities in Direct Oral Anticoagulant-Treated Patients

The increasing use of DOACs in patients with atrial fibrillation and other thromboembolic disorders has raised important considerations for AIS treatment. Current guidelines recommend IVT only if the last dose of DOAC was administered more than 48 h before symptom onset or if the anticoagulant effect is absent [2]. For dabigatran-treated patients, the specific reversal agent idarucizumab is recommended prior to IVT, while andexanet alfa is not endorsed for reversing factor Xa inhibitors in AIS patients [1].

Recent studies, however, have challenged these recommendations. A large international multicenter study found no significant increase in sICH in AIS patients who received IVT within 48 h of DOAC administration, regardless of the use of reversal agents or DOAC plasma level measurements [16]. Furthermore, studies suggest that DOAC-treated patients may experience better IVT outcomes and improved functional recovery compared to those not receiving anticoagulation [17,18].

Thrombi in patients treated with DOACs exhibit distinct structural and biochemical features compared to those in non-DOAC-treated individuals. A less compact fibrin network, thicker fibrin strands, and an increased proportion of white blood cells, likely resulting from the inhibition of thrombin-activatable fibrinolysis inhibitor [19,20], characterize these thrombi. Such alterations may render these thrombi more susceptible to thrombolysis, which could explain the enhanced efficacy of IVT observed in this patient population and the potential for improved outcomes with MT.

## 5. The Promise of Tenecteplase in Acute Ischemic Stroke

Overcoming resistance to rt-PA remains a significant challenge in the management of AIS. A promising approach to address this issue is the pharmacological targeting of non-fibrin thrombus components, such as platelets, NETs, and VWF, to enhance thrombolytic efficacy.

An alternative to rt-PA is TNK, a bioengineered variant of rt-PA, with three point-mutations that increase glycosylation and consequently reduce clearance and decrease interaction with PAI-1 [21], conferring notable pharmacokinetic and safety advantages. Compared with rt-PA, TNK exhibits higher fibrin specificity, enabling more targeted clot degradation while reducing systemic fibrinogen and plasminogen depletion [22,23], without significant disruption of the thrombolytic system of the former. This, in turn, may be associated with trends towards lower sICH incidence with TNK in the ATTEST trial [24]. Moreover, TNK is more resistant to PAI-1 and has a plasma half-life approximately five times longer than rt-PA (terminal/functional half-life reported ~20–25 min vs. ~4–6 min for rt-PA) and peak concentration achieved essentially immediately after the bolus, enabling easier application [22]. Administered as a single intravenous bolus over 5–10 s, TNK eliminates the need for a complex infusion regimen, reducing operational challenges, such as dosing errors and interruptions [23], considerably simplifying logistics, and even leads to shortened door-to-needle times (DNT) [25,26,27], all contributing to its safety and effectiveness in daily clinical practice.

Based on a comprehensive analysis of randomized controlled trials (RCTs), TNK at a 0.25 mg/kg dose is regarded as a valuable alternative to rt-PA for IVT in AIS demonstrating non-inferiority to rt-PA in broad populations [28,29,30,31,32,33,34,35,36] and superiority for excellent reperfusion in patients with LVOs [37] within 4.5 h of symptom onset. Interestingly, in patients with LVOs in anterior circulation 4.5 to 24 h after symptom onset selected upon CT perfusion (CTP) criteria, most of whom had undergone MT, TNK 0.25 mg/kg did not result in better clinical outcomes than those with placebo. However, sICH rate was similar in the two groups [38]. The 0.4 mg/kg dose is unsafe, as shown by significantly higher sICH rate and mortality in NOR-TEST 2 [39]. The safety profile of the 0.25 mg/kg dose is comparable to rt-PA, with consistent rates of sICH and mortality across different RCTs. TNK 0.25 mg/kg is non-inferior for general use, superior in LVOs, equally safe, and offers practical advantages, solidifying its role as a preferred thrombolytic agent. The key RCTs are presented in Table 2.

AcT: Alteplase compared to Tenecteplase; AIS: acute ischemic stroke; ATTEST: Alteplase versus tenecteplase for thrombolysis after ischaemic stroke; ATTEST-2: Alteplase versus tenecteplase for thrombolysis after ischaemic stroke; EXTEND-IA TNK: Tenecteplase versus Alteplase before Thrombectomy for Ischemic Stroke; CTP: CT perfusion; ITT: intention to treat; IVT: intravenous thrombolysis; NOR-TEST: Tenecteplase versus alteplase for management of acute ischaemic stroke; NOR-TEST 2: Tenecteplase versus alteplase for management of acute ischaemic stroke 2; RCT: randomized controlled trial; MT: mechanical thrombectomy; mRS: modified Rankin score; N: number of participants; NA: not available; NIHSS: National Institute of Health Stroke Scale; ORIGINAL: Tenecteplase vs. Alteplase for Patients With Acute Ischemic Stroke; PPA: per-protocol analysis; PPP: per-protocol population; rt-PA: alteplase; TASTE: Tenecteplase versus alteplase for thrombolysis in patients selected by use of perfusion imaging within 4·5 h of onset of ischaemic stroke; TNK: tenecteplase; TRACE: Safety and efficacy of tenecteplase versus alteplase in patients with acute ischaemic stroke; TRACE-2: Safety and efficacy of tenecteplase versus alteplase in patients with acute ischaemic stroke 2; vs.: versus.

Real-world evidence strongly corroborates RCT findings, showing a trend towards better functional outcomes with TNK [26,40,41,42,43,44], reaching statistical significance in some registries [45]. The sICH rate with TNK is comparable to rt-PA, and in some studies, it may be lower. A major and consistent finding is the significant reduction in DNT with TNK, directly attributable to its single-bolus administration. Real-world mortality rates are comparable between the two thrombolytics. TNK 0.25 mg/kg seems to be at least as effective and safe as rt-PA, while offering a significant logistical advantage for faster treatment. The key real-world studies are presented in Table 3.

## 6. Combination Therapies to Enhance Thrombolysis and Mechanical Thrombectomy

An emerging area of research is the potential for combination therapies to enhance thrombolysis and MT efficacy. One of the possible targets is the cerebral microcirculation with the “no-reflow” phenomenon despite a successful macrovascular recanalization in MT. The CHOICE trial suggested that an add-on IA rt-PA treatment was associated with higher rates of improved outcomes at 90 days [46]. Activated plasma VWF interacts with platelets and forming fibrin to produce thicker, more compact fibrin/platelet aggregates, reducing clot permeability and slowing diffusion of rt-PA/plasmin into the clot, making enzymatic lysis less efficient. Experimental work shows VWF can change fibrin polymerization and produce denser clots that are more resistant to thrombolysis [47]. Furthermore, VWF-mediated platelet recruitment concentrates platelet-derived PAI-1 within the thrombus, suppressing plasminogen activation. Inflammatory signals releasing VWF from endothelium often co-release or upregulate PAI-1, compounding thrombolysis resistance [48]. Based on the idea that VWF is a substantial component of platelet-rich thrombi, VWF has become a novel target for candidate treatments to improve thrombolysis [49]. Several strategies include the use of N-acetylcysteine (NAC), ADAMTS13 (a disintegrin and metalloproteinase with a thrombospondin type 1 motif, member 13), and agents that block the binding of platelets to VWF [49]. An ex vivo analysis of human stroke thrombi retrieved via EVT has demonstrated a significant reduction in thrombus weight when dimerized form of NAC is combined with rt-PA, specifically when the thrombus is platelet-rich [50].

Within blood vessels, NETs promote further thrombus formation and are associated with AIS and poorer outcomes [51]. Fibrin fibers become physically interwoven with NET DNA/histone filaments, producing a denser, mechanically stable clot that is less accessible to plasmin, this being a major reason for rt-PA resistance [52]. NET components bind plasminogen and rt-PA, lowering their effective concentration at the fibrin surface and slowing plasmin generation. A recent experimental study directly shows NETs can bind rt-PA/plasminogen and blunt rt-PA-induced lysis [53]. Core histones and other NET proteins alter fibrin structure and directly inhibit plasmin-mediated cleavage. Histones can bind fibrin (ogen) and plasmin (ogen), producing a bell-shaped modulation of plasmin generation but overall delay thrombolysis at pathologic concentrations; with consequent slower plasmin-driven fibrin degradation [54]. NETs activate platelets and endothelial cells, provoking secretion of PAI-1 from platelets and endothelium, thereby increasing local inhibition of plasmin generation [55].

There is a functional cross-talk between NETs and VWF, amplifying each other: NETs can trigger endothelial VWF release and platelet activation, while VWF-rich thrombi are better at trapping NETs—together creating a dense, antifibrinolytic composite [56]. The combined effects converge to produce hypofibrinolytic thrombi that are resistant to systemic rt-PA and slow to be cleared by plasmin. The degradation of NETs by DNase-1, a DNA-cleaving enzyme, shows promise as a novel approach to promoting arterial recanalization in AIS and is presently being studied in two studies, EXTEND-IA DNase and NETs-target [49]. Combining thrombolytic agents with adjunctive therapies targeting specific thrombus components and active processes may optimize treatment outcomes. For instance, pairing rt-PA with eptifibatide could inhibit platelet aggregation in platelet-rich thrombi [57], while rt-PA combined with argatroban may prevent fibrin deposition in fibrin-rich thrombi [58]. Furthermore, combining rt-PA with factor XI/XII inhibitors targeting NET-dependent coagulation or pairing rt-PA with ADAMTS-13 could facilitate the thrombolysis of VWF-rich, non-fibrin fibrillary thrombus components [5].

**Figure 1:**
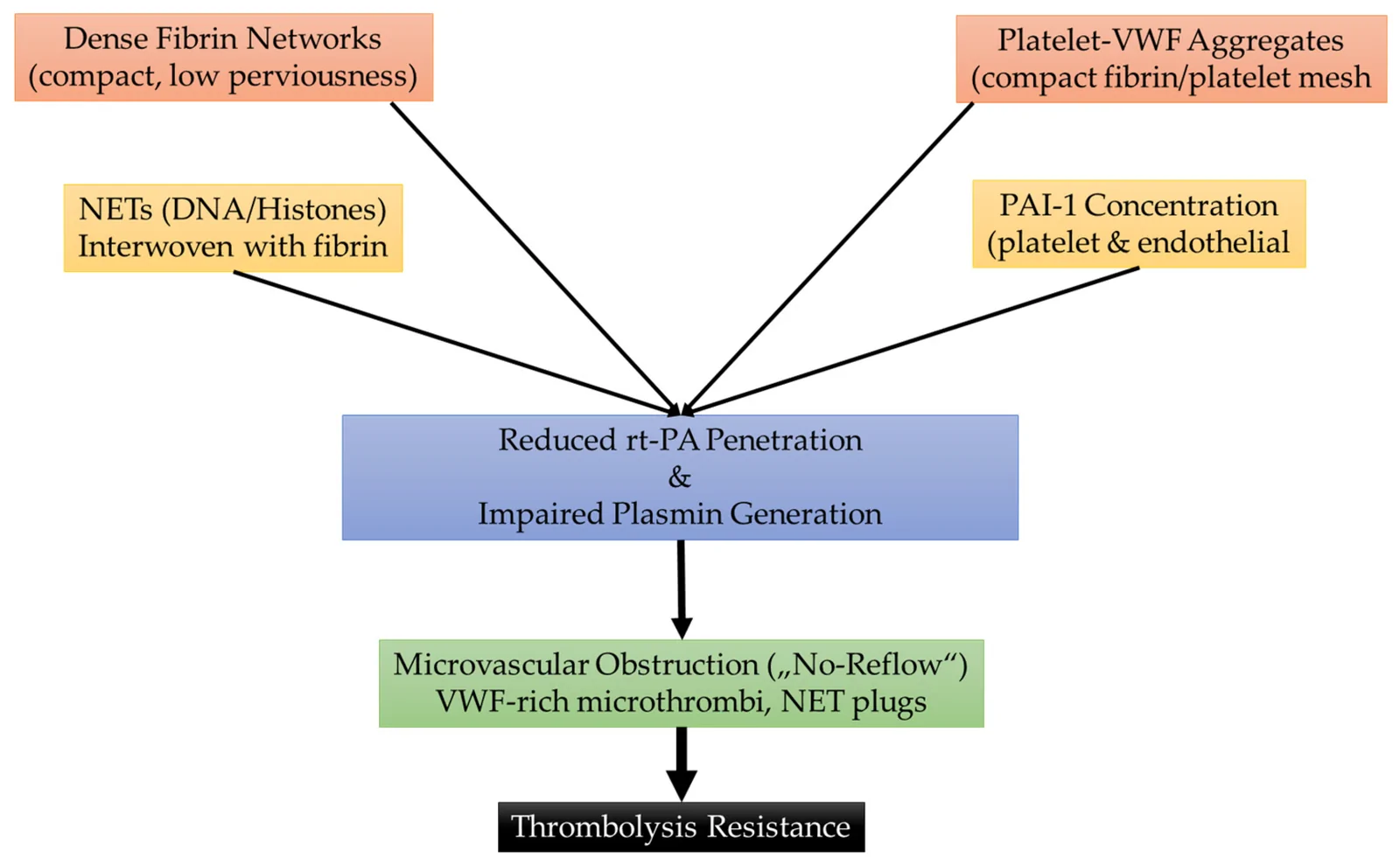
Key mechanisms of thrombolytic resistance.

By targeting multiple aspects of thrombus composition and coagulation, combination therapies may increase the effectiveness of IVT and MT, potentially improving outcomes in patients with complex thrombi and those resistant to standard treatments. We provide a summary of key mechanisms of thrombolytic resistance in Figure 1.

DNA: deoxyribonucleic acid; NET: neutrophil extracellular trap; PAI-1: plasminogen activator inhibitor 1; rt-PA: alteplase; VWF: von Willebrand factor.

## 7. Synergic Therapies to Achieve Reperfusion

The recent introduction of TNK offers an unprecedented opportunity to analyze the optimal interaction between thrombolytic therapy and MT in AIS. While the combination of IVT using rt-PA and MT has demonstrated potential benefits, the current level of evidence remains insufficient. Some studies showed no advantage of associating IVT to MT [59,60], while others failed to demonstrate superiority or non-inferiority of MT alone vs. MT associated with intravenous (IV) rt-PA [61,62]. A meta-analysis confirmed that all studies designed as non-inferiority studies remained underpowered to establish the superiority of associating IVT and MT [63].

An important factor influencing these findings is the role of time. Further analysis of existing data indicates that the benefit of combining rt-PA with MT diminishes significantly beyond a time window of 2 h and 20 min, suggesting a critical temporal threshold for this therapeutic synergy [64].

Another limitation of IVT with rt-PA lies in the historical lack of precision in assessing its efficacy. When rt-PA was first implemented, imaging tools were insufficient to demonstrate the presence of thrombi, their location, or the extent of hypoperfusion. Stroke diagnosis relied on clinical suspicion without the robust imaging modalities now available.

Since TNK has been introduced in a revolutionized landscape with all imaging modalities accessible at a large scale, a more accurate evaluation of its efficacy, encompassing not only clinical outcomes but also imaging-based assessments of thrombus presence, type, location, recanalization rates, and time to thrombus resolution, is feasible. This broader evaluation may enable efficacy to be defined by recanalization rates in correlation with thrombus characteristics, time for thrombus to resolve and time from symptom onset to IV TNK administration.

Further research is essential to refine these criteria and elucidate how TNK can best interact with MT. For instance, it remains unclear whether TNK enhances the mechanical retrieval of thrombi or if its primary benefit lies in dissolving smaller thrombi that fragment and migrate distally during MT. Importantly, future studies should move beyond non-inferiority designs when evaluating novel thrombolytic agents like TNK in combination with MT. Scenarios in which combining TNK with MT might cause more harm than either treatment alone must also be carefully considered.

Given TNK’s unique pharmacological properties, its mode of administration may warrant reconsideration. Evidence from the PROACT-II trial, which reported a 10% intracranial hemorrhage rate with IA urokinase, curtailed the widespread adoption of IA fibrinolytic delivery. However, TNK’s higher fibrin specificity and longer half-life may make it particularly effective against fibrin- and platelet-dominant clots that are both resistant to rt-PA and challenging to extract mechanically. IA administration of TNK, alone or in combination with IV therapy, could offer significant advantages for these thrombus subtypes [65]. Prospective studies are needed to assess the safety and efficacy of such approaches, as well as the potential to reduce hemorrhagic risk through optimized delivery strategies.

## 8. Future Perspectives

The management of AIS is rapidly advancing, particularly with innovations in thrombolytic therapies and MT. Despite advances in reperfusion treatment, resistance to recanalization persists in a substantial proportion of patients, largely due to thrombus heterogeneity and delayed intervention. A key element in optimizing treatment strategies is understanding the composition of stroke thrombi and how they respond to different therapeutic interventions. RBC-rich thrombi, typically less compact, respond favorably to both IVT and MT, while fibrin/platelet- or NET-dense clots are more resistant. They often require multiple thrombectomy passes and are associated with worse outcomes.

On the one hand, the growing population of patients treated with DOACs introduces additional complexity mirrored in the existing guidelines [1], but on the other hand, DOAC-associated thrombi appear looser and more lysis-prone, which may explain favorable responses to IVT in the existing non-randomized trials [10,11]. Further RCTs are mandatory to establish the safety and efficiency of these novel strategies that would support potential reconsideration of existing eligibility criteria for IVT in DOAC-treated patients.

**Figure 2:**
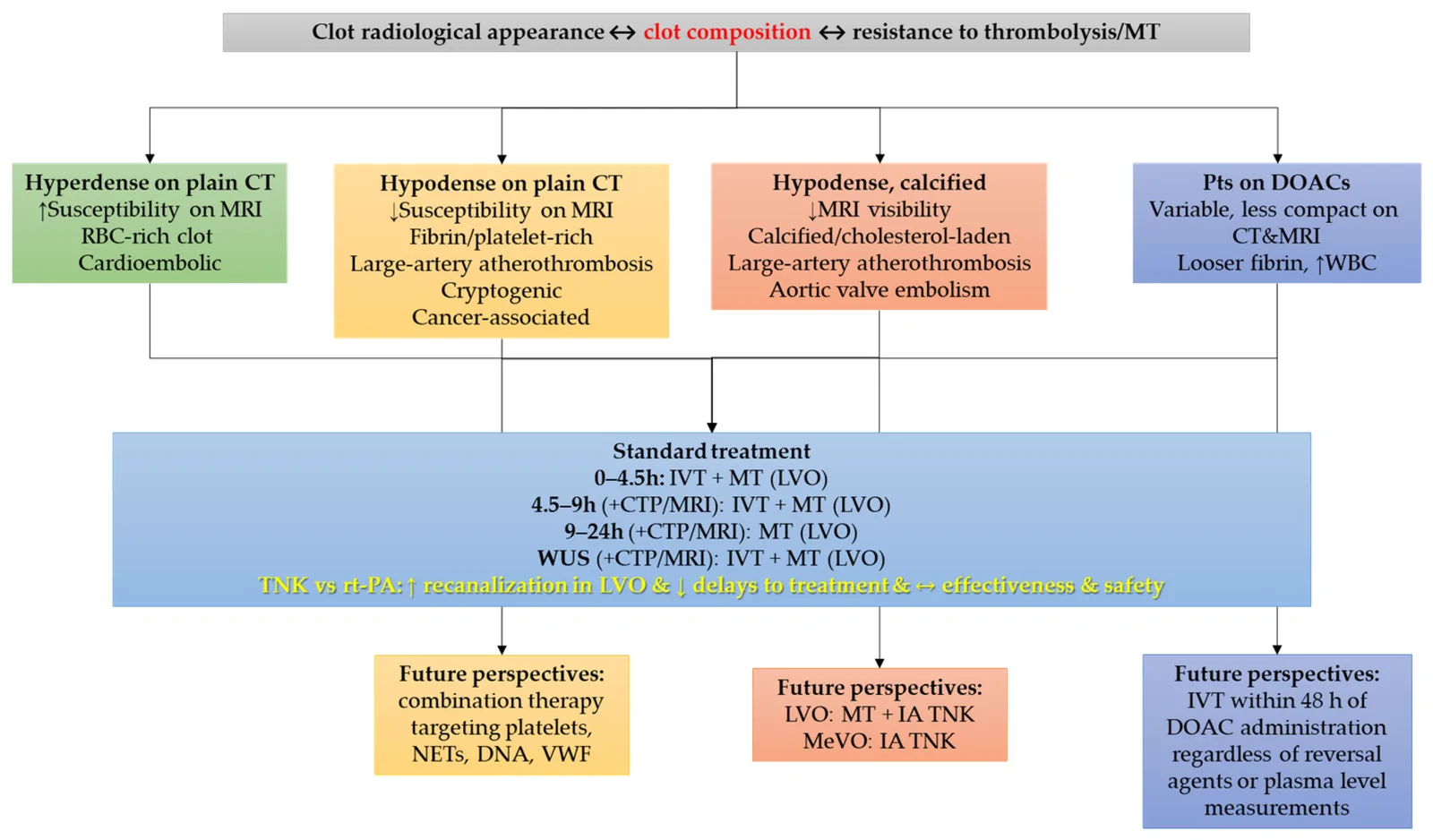
A practical stratification algorithm based on clot composition and evolving strategies in acute ischemic stroke. ↓: denotes “lower”; ↑: denotes “higher”; CT: computerized tomography; CTP: computerized tomography perfusion; DOAC: direct oral anticoagulant; DNA: deoxyribonucleic acid; IA: intra-arterial; IVT: intravenous thrombolysis; MeVO: medium vessel occlusion; MRI: magnetic resonance imaging; MT: mechanical thrombectomy; NETs: neutrophil extracellular traps; pts: patients; rtPA: alteplase; TNK: tenecteplase; vs.: versus; VWF: von Willebrand factor; WBC: white blood cell; WUS: wake-up stroke.

TNK presents several notable advantages over rt-PA, including a simpler administration process, improved pharmacokinetics, and promising efficacy in both MT and IA applications. Clinical data demonstrate that TNK is at least non-inferior to rt-PA with trends toward better functional outcomes and fewer hemorrhagic complications. Moreover, IA TNK as an adjunct to MT may further enhance recanalization and clinical recovery, particularly in fibrin- or platelet-dominant thrombi. The evolving role of TNK within multimodal reperfusion strategies warrants further exploration. Advanced imaging now allows detailed assessment of thrombus biology and microvascular perfusion, enabling precise evaluation of how TNK interacts with MT or adjunctive agents. Whether TNK enhances mechanical retrieval or preferentially dissolves distal emboli remains to be clarified. Future trials should move beyond non-inferiority frameworks to establish the optimal timing, route, and combination of TNK-based therapies for diverse thrombus phenotypes. Furthermore, the exploration of combination therapies targeting non-fibrin components of thrombi holds great potential to enhance the effectiveness of thrombolysis and improve patient outcomes. By addressing multiple aspects of thrombus structure and coagulation dynamics, such therapies could offer solutions for patients with thrombi resistant to standard treatments. Figure 2 shows a proposed stratification algorithm based on putative clot composition, influencing treatment resistance and consequently, evolving therapies in AIS.

## 9. Conclusions

Modern AIS management is entering an era of precision thrombolysis, in which therapy selection is guided by thrombus composition, patient profile, and timing. Tenecteplase, with its favorable pharmacodynamics and compatibility with mechanical and adjunctive approaches, represents a cornerstone for this next phase of individualized reperfusion therapy. As the field progresses, further clinical trials and research will be necessary to refine treatment protocols, especially for challenging patient groups, such as those on DOACs or that experiencing thrombolysis resistance. These ongoing studies are essential for ensuring that AIS patients receive the most effective and personalized therapies to optimize outcomes.

## Figures and Tables

**Table 1:** Summary of thrombus composition, imaging correlates, and treatment response in acute ischemic stroke.

Thrombus Type	Key Features	Imaging Correlates	Treatment Response
RBC-rich	Loose fibrin network, high RBC content	Hyperdense artery sign on CT; susceptibility vessel sign on MRI	Good response to IVT and MT; often easier retrieval
Fibrin/Platelet-rich	Dense fibrin, abundant platelets, intermeshed NETs/DNA	Low-density clot on CT; less prominent susceptibility on MRI	Poor IVT response; resistant to MT, often requires multiple passes
Calcified/Cholesterol-laden	Rigid structure, calcium or cholesterol crystals present	Hypodense/calcified appearance on CT; poor visibility on MRI	Highly resistant to IVT and MT; difficult or incomplete retrieval
In patients on DOACs	Looser fibrin network, thicker strands, ↑ WBC content	Variable; may appear less compact on CT/MRI	Potentially more susceptible to IVT; favorable outcomes observed in MT

CT: computerized tomography; DNA: deoxyribonucleic acid; IVT: intravenous thrombolysis; MRI: magnetic resonance imaging; MT: mechanical thrombectomy; NETs: neutrophil extracellular traps; RBC: red blood cell; WBC: white blood cell; ↑: denotes “higher”.

**Table 2:** Key randomized controlled studies of tenecteplase vs. alteplase in acute ischemic stroke.

RCT (Year)	Phase	N	TNK Dose	Key Population	Primary Outcome (mRS 0–1 at 90 d Unless Stated)	Functional Outcome (TNK vs. rt-PA) mRS 0–1	sICH	Mortality	DNT (Min, Median)	Conclusion
Parsons et al. (2012) [28]	2	75	0.1 and 0.25 mg/kg	AIS with perfusion lesion within 6 h of symptom onset, no MT	Reperfusion at 24 h: 79% vs. 55% (*p* = 0.004) NIHSS improvement at 24 h: ↓ 8 vs. ↓ 3	54% vs. 40% (*p* = 0.25)	4% vs. 12% (*p* = 0.33)	8% vs. 12% (*p* = 0.68)	NA	TNK with significantly better reperfusion and NIHSS improvement compared with rt-PA
ATTEST (2015) [29]	2	104	0.25 mg/kg	AIS within 4.5 h of symptom onset, no MT	% penumbral salvage: 68% in either group (*p* = 0.81)	28% vs. 20% (*p* = 0.28)	2% vs. 4% (*p* = 0.55)	17% vs. 12% (*p* = 0.51)	42 vs. 38	TNK and rt-PA with similar neurological and neuroradiological outcomes
NOR-TEST (2017) [30]	3	1100	0.4 mg/kg	AIS (mild, median NIHSS 4) within 4.5 h of symptom onset or 4.5 h of awakening with symptoms, MT allowed	mRS 0–1 at 90 d	64% vs. 63% (*p* = 0.52)	3% vs. 2% (*p* = 0.70)	5% vs. 5% (*p* = 0.68)	32 vs. 34	TNK not superior to rt-PA &similar safety
EXTEND-IA TNK (2018) [37]	2	202	0.25 mg/kg	AIS with LVOs within 4.5 h of symptom onset, MT-eligible pts	Reperfusion > 50% on initial angiogram: TNK superior to rt-PA for excellent reperfusion (22% vs. 10%) (*p* = 0.002 for noninferiority; *p* = 0.03 for superiority)	51% vs. 43% (*p* = 0.23)	1% vs. 1% (*p* = 0.99)	10% vs. 18% (*p* = 0.08)	NA	TNK superior to rt-PA in restoring perfusion in the territory of a proximal-cerebral artery occlusion
TRACE (2022) [31]	2	236	0.1, 0.25, 0.32 mg/kg	AIS within 3 h of symptom onset in Chinese, MT allowed but excluded from PPA	NIHSS ↓ ≥ 4 or ≤1 at day 14: TNK 0.1 mg: 63% vs. TNK 0.25 mg: 77% vs. TNK 0.32 mg: 67% vs. rt-PA: 63%	TNK 0.1 mg: 55% vs. TNK 0.25 mg: 64% vs. TNK 0.32 mg: 62% vs. rt-PA: 59%	TNK 0.1 mg: 5% vs. TNK 0.25 mg: 0% vs. TNK 0.32 mg: 3.3% vs. rt-PA: 1.7 (*p* = 0.52)	TNK 0.1 mg: 10% vs. TNK 0.25 mg: 1.8% vs. TNK 0.32 mg: 8.3% vs. rt-PA: 10.2%	TNK 0.1 mg: 71 vs. TNK 0.25 mg: 60 vs. TNK 0.32 mg: 69 vs. rt-PA: 71	TNK at all doses well tolerated
NOR-TEST 2 (2022) [39]	3	204	0.4 mg/kg	Moderate-severe AIS (NIHSS ≥ 6) within 4.5 h of symptom onset, MT allowed	mRS 0–1 at 90 d	32% vs. 51% (*p* = 0.0064)	6% vs. 1% (*p* = 0.061)	16% vs. 6% (*p* = 0.013)	NA	TNK 0.4 mg/kg worse safety and functional outcomes compared to rt-PA 0.9 mg/kg
AcT (2022) [32]	3	1577	0.25 mg/kg	AIS within 4.5 h of symptom onset meeting standard IVT criteria, MT allowed	mRS 0–1 at 90 d	37% vs. 35%	3.4% vs. 3.2%	15.3% vs. 15.4%	36 vs. 37	TNK reasonable alternative to rt-PA
TRACE-2 (2023) [33]	3	1417	0.25 mg/kg	Moderate-severe AIS (NIHSS 5-25) within 4.5 h of symptom onset, Chinese, ineligible for MT	mRS 0–1 at 90 d	62% vs. 58%	2% vs. 2%	7% vs. 5%	58 vs. 61	TNK non-inferior to rt-PA
ORIGINAL (2024) [34]	3	1465		AIS, Chinese, within 4.5 h of symptom onset, MT allowed	mRS 0–1 at 90 d	73% vs. 70%	1.2% vs. 1.2%	4.6% vs. 5.8%	NA	TNK non-inferior to rt-PA, similar safety
TASTE (2024) [35]	3	680		AIS, within 4.5 h of symptom onset, selected by CTP, no MT	mRS 0–1 at 90 d	57% vs. 55% (*p* = 0.031 for non-inferiority)	3% vs. 2%	7% vs. 4%	65 vs. 64	TNK non-inferior to rt-PA in PPP, not in ITT analysis
ATTEST-2 (2024) [36]	3	1777	0.25 mg/kg	Moderate-severe AIS (NIHSS ≥ 6) within 4.5 h of symptom onset, MT allowed	mRS 0–1 at 90 d	44% vs. 42% (*p* = 0.0018 for non-inferiority, 0.40 for superiority)	2% vs. 2% (*p* = 0.37)	8% vs. 8% (*p* = 0.80)	47 vs. 46	TNK non-inferior to rt-PA

↓: denotes “lower”.

**Table 3:** Key real-world studies of tenecteplase vs. alteplase in acute ischemic stroke.

RWS (Year)	Design	N (TNK/rt-PA)	Functional Outcome (mRS 0–2 at 90 d Unless Stated)	sICH Rate	Mortality	DNT (Min)	Conclusion
Tsivgoulis et al. (2022) [45]	Prospectively collected data from SITS-ISTR	331/797 MT: 7%/5% PSMG	68% vs. 52% (*p* < 0.001)	1.0% vs. 1.3%	11% vs. 23% (*p* < 0.001)	158 vs. 158 (OTT)	TNK with better outcomes, no increased risk of sICH
Swedish Stroke Register (2024) [40]	Retrospective registry-based	888/6560 MT: 15%/17.5%	53% vs. 51%	5% vs. 4.4%	14% vs. 12%	34 vs. 43	TNK not non-inferior to rt-PA in safety; ↓ DNT (−9 min)
Murphy et al. (2023) [41]	Retrospective US cohort from 54 academic centers	3432/55,894 MT: not reported	NA	0.3% vs. 1.4% of major bleeding, requiring blood transfusion ICH: (3.5% vs. 3.0%)	8.2% vs. 9.8%	NA	TNK with ↓ mortality, ICH and blood loss
Yao et al. (2024) [42]	Single-center retrospective observational cohort	120/144 MT: 20%/23%	NIHSS improvement at 24 h: 64% vs. 50% (*p* = 0.024), length of hospitalization: 6 d vs. 8 d	3.3% vs. 4.9%	NA	36.5 vs. 50	TNK with significant reduction in DNT (−13.5 min) and early NIHSS improvement
Zhao et al. (2024) [43]	Single-center retrospective observational cohort	79/147 MT: 28%/33%	58% vs. 51% (*p* = 0.37)	2.5% vs. 6.1% (*p* = 0.38)	14% vs. 10%	43 vs. 43	TNK with comparable safety and functional outcomes
Sekita et al. (2025) [26]	Single-center retrospective observational cohort	138/138 MT: 78%/73%	54% vs. 43%	2% vs. 1%	5% vs. 9% (in-hospital)	27 vs. 34	TNK with comparable outcomes at discharge and shorter DNT (−7 min)
Rousseau et al. (2025) [44]	Prospective data from Get With The Guidelines–Stroke registry	9465/70,085 MT: 17.7%/13.8%	45% vs. 46% (at discharge)	3.1% vs. 3.1%	5% vs. 4.6% (in-hospital)	LKW-IVT: 120 vs. 124	TNK with similar safety and effectiveness outcomes to rt-PA

DNT: door-needle time; ICH: intracerebral hemorrhage; IVT: intravenous thrombolysis; mRS: modified Rankin Score; LKW: last-known well; MT: mechanical thrombectomy; N: number of participants; NA: not available; NIHSS: National Institute of Health Stroke Scale; OTT: onset-treatment time; PSMG: propensity score matched groups; rt-PA: alteplase; RWS: real-world study; sICH: symptomatic intracranial hemorrhage; SITS-ISTR: Safe Implementation of Thrombolysis in Stroke-Thrombolysis Register; TNK: tenecteplase; US: United States; ↓: denotes “lower”.

## Data Availability

No new data were created or analyzed in this study. Data sharing is not applicable to this article.

## Abbreviations

The following abbreviations are used in this manuscript:

AIS	Acute ischemic stroke
CT	Computerized tomography
CTA	Computerized tomography angiography
CTP	Computerized tomography perfusion
DNA	Deoxyribonucleic acid
DNT	Door-needle time
DOAC	Direct oral anticoagulant
GRE	Gradient recalled echo
IA	Intra-arterial
IV	Intravenous
IVT	Intravenous thrombolysis
LVO	Large vessel occlusion
MRI	Magnetic resonance imaging
MT	Mechanical thrombectomy
NET	Neutrophil extracellular trap
PAI-1	Plasminogen activator inhibitor 1
PET	Positron emission tomography
RBCs	Red blood cells
RCT	Randomized controlled trial
rt-PA	Alteplase
sICH	Symptomatic intracerebral hemorrhage
SWI	Susceptibility-weighted imaging
TNK	Tenecteplase
VWF	Von Willebrand factor
